# Levodopa–entacapone–carbidopa intestinal gel infusion in advanced Parkinson’s disease: real-world experience and practical guidance

**DOI:** 10.1177/17562864221108018

**Published:** 2022-06-26

**Authors:** Dag Nyholm, Wolfgang H. Jost

**Affiliations:** Professor of Neurology, Department of Medical Sciences, Neurology, Uppsala University, 75185 Uppsala, Sweden; Parkinson-Klinik Ortenau, Wolfach, Germany

**Keywords:** dosing, levodopa–entacapone–carbidopa intestinal gel infusion, motor symptoms, Parkinson’s disease, patient-reported outcomes, safety, tolerability

## Abstract

As Parkinson’s disease (PD) progresses, treatment needs to be adapted to maintain symptom control. Once patients develop advanced PD, an optimised regimen of oral and transdermal medications may no longer provide adequate relief of OFF periods and motor complications can emerge. At this point, patients may wish to consider a device-aided therapy (DAT) that provides continuous dopaminergic stimulation to help overcome these issues. Levodopa–entacapone–carbidopa intestinal gel (LECIG) infusion is a recently developed DAT option. The aim of this article is twofold: (1) to give an overview of the pharmacokinetics of LECIG infusion and clinical experience to date of its use in patients with advanced PD, including real-world data and patient-reported outcomes from a cohort of patients treated in Sweden, the first country where it was introduced, and (2) based on that information to provide practical guidance for healthcare teams starting patients on LECIG infusion, whether they are transitioning from oral medications or from other DATs, including recommendations for stepwise dosing calculation and titration. In terms of clinical efficacy, LECIG infusion has been shown to have a similar effect on motor function to standard levodopa–carbidopa intestinal gel (LCIG) infusion but, due to the presence of entacapone in LECIG, the bioavailability of levodopa is increased such that lower overall levodopa doses can be given to achieve therapeutically effective plasma concentrations. From a practical standpoint, LECIG infusion is delivered using a smaller cartridge and pump system than LCIG infusion. In addition, for patients previously treated with LCIG infusion who have an existing percutaneous endoscopic transgastric jejunostomy (PEG-J) system, this is compatible with the LECIG infusion system. As it is a relatively new product, the long-term efficacy and safety of LECIG infusion remain to be established; however, real-world data will continue to be collected and analysed to provide this information and help inform future clinical decisions.

## Introduction

The progressive nature of Parkinson’s disease (PD) presents many challenges for effective clinical management. With a heterogeneous and changing clinical picture as the disease advances, there is a need to regularly review and individually adapt treatment to adequately manage each patient’s motor and nonmotor symptoms, and ensure they can maintain the best possible quality of life.

Oral levodopa is the mainstay of initial therapy for PD, but the response to oral levodopa changes as PD advances due to the gradual loss of dopaminergic neurons in the brain.^1^ As a result, achieving a good clinical response to oral levodopa becomes less frequent (a narrowing therapeutic window), and at this point, adjunctive oral/transdermal treatment options may need to be considered to try and make sure the patient’s symptoms are adequately controlled. Following a diagnosis of PD, there is generally a relatively short period where the effects of oral levodopa and adjunctive medications are stable; however, eventually motor complications (motor fluctuations and dyskinesias) can develop and the patient is considered to have ‘advanced’ disease^2^ – this is generally the larger proportion of the patient’s time living with PD. Motor complications can arise from either inadequate dopaminergic stimulation resulting in substantial ‘OFF’ time or excess dopaminergic stimulation resulting in dyskinesias,^3^ and if these are insufficiently treated, it can have a significant impact on patients’ quality of life,^4–6^ that of their caregivers,^7,8^ and also on healthcare system resources.^6,7,9,10^

Two recent studies undertaken in Sweden have highlighted the significant healthcare resource and societal costs of managing later stage and advanced PD, and importantly the detrimental effect on patients’ health-related quality of life living with advancing disease.^6,10^ With increasing OFF time, total care costs were found to increase while patients’ productivity and ability to work decreased,^6^ emphasising the importance of timely and effective symptom management.

Various strategies have been employed in an effort to overcome the therapeutic challenges that arise as PD advances with the aim of improving the delivery and extend the clinical effect of oral PD medication. These include the use of slow-release formulations, fractionation of levodopa doses throughout the day, frequent administration of levodopa/carbidopa microtablets,^11,12^ or the use of adjunctive oral medications, such as dopamine agonists, catechol-O-methyltransferase (COMT) inhibitors and monoamine oxidase type-B (MAO-B) inhibitors.^13^

Treatment choices in PD can be further complicated if the patient has gastrointestinal issues, such as delayed gastric emptying or swallowing difficulties, which are common in PD and can impact the clinical effect of oral medication,^14^ highlighting the need for therapies that can bypass this route.^15^ Subcutaneous apomorphine pen injection is an adjunctive, nonoral, ‘on-demand’ option that can be employed to provide a rapid and reliable return to the ‘ON’ state for those patients experiencing ‘OFF’ episodes, such as early-morning OFF periods, that can still occur between doses of optimised oral medication.^16,17^

However, whatever the formulation or dosing strategy, oral medications and most adjunctive therapies are administered intermittently, resulting in pulsatile stimulation of dopamine receptors.^18^ Continuous dopaminergic stimulation (CDS) has therefore been proposed as an alternative and more physiological approach to the delivery of PD medication that can potentially reduce the risk of developing motor complications.^19,20^ This has led to the development of a range of effective device-aided therapies (DATs) that can provide CDS when optimised oral/transdermal PD therapy is no longer able to adequately control motor symptoms.

This publication focuses on the most recently available device-aided therapy (DAT), levodopa–entacapone–carbidopa intestinal gel (LECIG) infusion (Lecigon^®^/Lecigimon^®^; Lobsor Pharmaceuticals AB, Uppsala, Sweden). The primary objective of this article is to provide an overview of the pharmacokinetic and clinical data available to date in patients with advanced PD. This includes clinical experience, real-world data and patient-reported outcomes with LECIG infusion treatment in countries where it has been launched. Based on this information, the secondary objective is to provide practical guidance for healthcare teams starting patients on LECIG infusion.

## Options for the delivery of continuous dopaminergic stimulation

Currently available DAT options for advanced PD management comprise deep brain stimulation or continuous infusions of apomorphine (subcutaneously) or levodopa (directly into the upper jejunum). At the present time, no prospective, head-to-head studies have been performed to compare these therapies; however, all these treatment options have been shown in individual clinical studies to have broadly similar efficacy and to be relatively well tolerated, albeit with different adverse event profiles.^21^ This does not mean that every option is suitable for every PD patient and careful matching of each individual with the most suitable therapy for their particular clinical picture, personal circumstances and preferences is critical. Patients considering a DAT should be provided with information about all available options so they can make an informed choice in consultation with their neurologist and maximise the success of the chosen therapy.

An analysis of prescription of DATs in Sweden published in 2021 found that while their use has increased in Sweden since evidence-based treatment guidelines were published in 2016, it is still not at the levels suggested in these recommendations.^22^

### Deep brain stimulation

Deep brain stimulation (DBS) requires brain surgery to insert an electrode into the subthalamic nucleus (the current standard) or the globus pallidus interna (especially for dystonia or dyskinesia), or the nucleus ventralis intermedius (especially for tremor). High-frequency stimulation of these has been shown to provide long-term improvement in motor fluctuations and dyskinesias in patients with advanced PD.^23^

Although it is generally used for management of advanced PD, the Early-STIM study evaluated DBS in patients with less advanced disease where it was shown to be superior to conventional ‘best medical therapy’ in terms of motor disability, activities of daily living, motor complications and time with good mobility and no dyskinesia.^24^

### Subcutaneous apomorphine infusion

Subcutaneous apomorphine infusion is the least invasive of the available DATs and currently the only nonsurgical option. Numerous short-term and long-term uncontrolled studies have shown the efficacy of apomorphine infusion in reducing OFF time, with reductions of up to 80% reported, and most have also shown an improvement in dyskinesias and concomitant reductions in oral levodopa doses.^25^ The TOLEDO study was the first randomised, placebo-controlled, multicentre study of subcutaneous apomorphine infusion in patients with persistent motor fluctuations despite taking optimised oral medication and comprised a 12-week double-blind phase followed by a 52-week open-label phase.^26,27^ The results showed a significant and enduring reduction in OFF time accompanied by a significant improvement in ON time without troublesome dyskinesia, as well as a reduction in the daily dose and the number of doses of short-acting oral antiparkinsonian medication.

### Levodopa–carbidopa intestinal gel infusion

Levodopa–carbidopa intestinal gel (LCIG) infusion is delivered directly to the proximal jejunum following surgical placement of a percutaneous endoscopic gastrostomy tube with a jejunal extension (PEG-J) and is delivered *via* a portable, programmable infusion pump. Evidence for the efficacy and tolerability of LCIG has been demonstrated in several prospective clinical trials.^28^ A 12-week double-blind comparison of LCIG with standard levodopa therapy, a 52-week open-label study extension of the double-blind study, and a 54-week open-label safety study showed significant improvements in ‘OFF’ time and ‘ON’ time without troublesome dyskinesia, and quality of life (QoL) which were sustained with long-term use.^28^ A systematic literature review of data on the long-term effects of LCIG therapy (⩾12 months) found that it provided a durable effect in reducing OFF-time.^29^

### LECIG infusion

The most recent addition to the available DAT options is LECIG infusion. In terms of its method of delivery, it is very similar to LCIG infusion and uses a PEG-J system but with a smaller, specially designed, lightweight pump, the Crono^®^ LECIG pump (Canè, Turin, Italy). The formulation includes the COMT inhibitor, entacapone. Oral entacapone is sometimes given alongside LCIG infusion but requires multiple daily doses, whereas LECIG infusion combines the three products in one gel for infusion. Due to the presence of entacapone, the bioavailability of levodopa from LECIG infusion is higher than from LCIG infusion, and therefore reduced overall daily doses of levodopa can be given to achieve the same effective and stable plasma levodopa levels.^30,31^ LECIG infusion was first approved for use in Sweden in 2018 and has now received marketing authorisation in several other European countries.

## Levodopa metabolism and the development of levodopa-based therapies

Before focusing on LECIG infusion specifically, it is useful to review how knowledge of the pathways of levodopa metabolism and pharmacokinetics has led to the development of various levodopa drug formulations that aim to extend its bioavailability and improve its clinical efficacy. Levodopa itself was first isolated in 1911, introduced as a treatment for PD in the 1960s,^32^ and is still recognised today as the ‘gold standard’ PD therapy. In the peripheral tissues, levodopa can be metabolised to either dopamine (by decarboxylation) or other metabolites (by methylation). Decarboxylation of levodopa to dopamine is catalysed by dopa decarboxylase (DDC) while methylation is catalysed by COMT that results in the production of 3-O-methyldopa (3-OMD) and S-adenosyl-homocysteine, which is further metabolised to homocysteine.

Inhibition of these metabolic pathways in the peripheral tissues is desirable as this will maximise the amount of levodopa available to cross the blood–brain barrier and exert a clinical effect. In the early 1970s, the clinical advantages of adding a DDC inhibitor (DDCI), such as carbidopa or benserazide, to oral levodopa, were identified. Combination of a DDCI with levodopa was found to be associated with reduced side effects and better symptom control compared with levodopa alone, and these combinations are now used routinely in clinical practice. The first of these, levodopa–carbidopa, became commercially available in 1975,^33^ followed subsequently by levodopa–benserazide.

In 1998, the COMT inhibitor entacapone was approved by the European Medicines Agency (EMA) in Europe for oral use in combination with levodopa–carbidopa or levodopa–benserazide, thereby also allowing inhibition of the methylation pathway.^34^ Other COMT inhibitors have been introduced – tolcapone in 1997^35^ and opicapone in 2016.^36^ Tolcapone was later voluntarily withdrawn from the European Union (EU) market in 1998 due to reports of hepatotoxicity, but reinstated in 2004 as further follow-up showed that severe liver injury was an extremely rare event.^37^ In addition to extending the bioavailability of levodopa, an additional advantage of inhibiting peripheral levodopa metabolism with a COMT inhibitor is that some of the metabolites that result from methylation, namely, 3-OMD and homocysteine, are reduced, which may be of interest. The half-life of 3-OMD is approximately 15 h, which is longer than that of levodopa at about 1 h, so it can accumulate in the plasma and the brain.^38^ Sustained elevated levels of 3-OMD are therefore likely to increase the levels of other metabolites in the pathway, such as homocysteine.^39^ Elevated levels of homocysteine are known to be associated with a range of adverse events, including development of peripheral neuropathy (dysfunction of the sensory, motor and/or autonomic nerves), and with vascular risk factors (e.g. coronary heart disease, atherosclerosis and stroke).^39,40^ Although associations between increased levels of these metabolites with a range of adverse effects are well documented, whether they are causative factors still remains to be determined.

In the 1990s, the first infusion of a gel containing levodopa and carbidopa delivered directly into the upper jejunum (LCIG infusion) was developed at Uppsala University, Sweden, aiming to take advantage of the more stable plasma levodopa levels achieved when delivering continuous dopaminergic stimulation. It was approved initially by the Swedish Medical Products Agency, in 2004, followed by the European Medicines Agency for the rest of Europe in 2005, and then the US Food and Drug Administration in 2015.^41^

While levodopa is widely acknowledged as the mainstay of PD therapy, long-term exposure to levodopa may be associated with the development of peripheral neuropathy. Peripheral neuropathy has been frequently reported in Parkinson’s patients receiving prolonged treatment with both oral and intestinal infusion of levodopa.^39,42,43^ Neuropathy is thought to be linked to the duration of levodopa exposure,^44^ the use of high doses of levodopa,^45–47^ and the presence of high levels of plasma homocysteine.^40,45^ LCIG infusion has proved to be a beneficial treatment option for advanced PD but it involves an invasive surgical procedure and has been associated with both surgical and postsurgical complications, device problems, and other adverse events, including, as mentioned, peripheral neuropathy.^48^ These are important factors to consider when selecting treatment for PD patients and highlight the importance of ongoing monitoring and vigilance for adverse events.

## Characteristics of LECIG infusion

**Figure 1.**
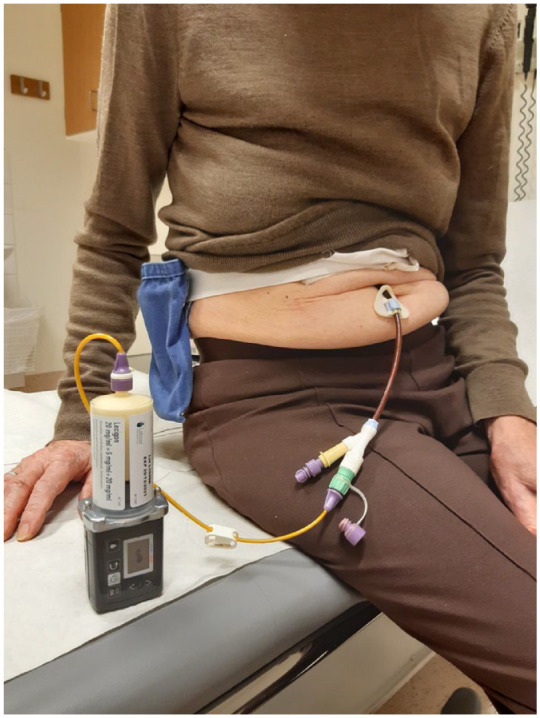
A Parkinson’s patient using LECIG infusion.

Previous studies have shown that combining LCIG infusion with an oral COMT inhibitor enables the dose of LCIG to be reduced by at least 20% while still maintaining stable levodopa concentrations and motor function.^49–51^ These findings led to the development of LECIG infusion in which three established PD therapies – levodopa, the DDCI carbidopa and the COMT inhibitor entacapone – are combined in a single gel formulation, negating the need to take a separate oral COMT inhibitor. Entacapone was selected as the COMT inhibitor in LECIG rather than tolcapone due to its better safety profile and lack of association with hepatotoxicity; opicapone was not available when LECIG was initially developed. LECIG is supplied in 47 ml cartridges each containing 940 mg levodopa (20 mg/ml), 235 mg carbidopa monohydrate (5 mg/ml) and 940 mg entacapone (20 mg/ml) and is indicated for treatment of advanced PD with severe motor fluctuations and hyperkinesia or dyskinesia when available oral combinations of Parkinson’s medication have not given satisfactory clinical efficacy. LECIG is delivered using the specially designed Crono^®^ LECIG pump that measures 55 × 150 mm and has a total weight of 227 g (Figure 1).

## Pharmacokinetics, clinical effects and tolerability of LECIG infusion

**Figure 2.**
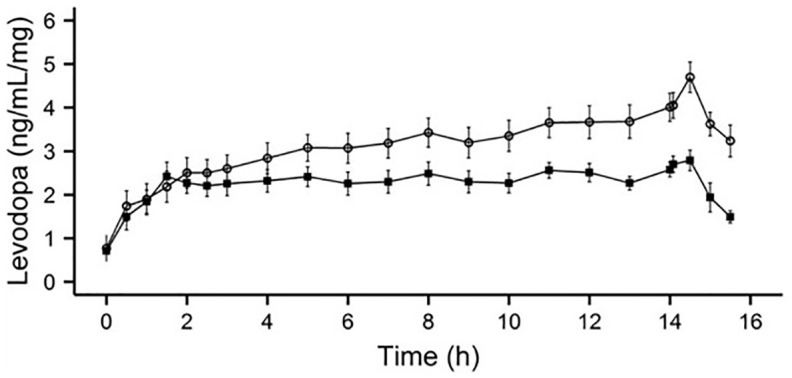
Pharmacokinetic mean (±SE) dose-adjusted plasma concentrations of levodopa (LCIG shown as filled squares; LECIG shown as open circles).^30^ Reproduced with permission from *Movement Disorders*.

**Figure 3.**
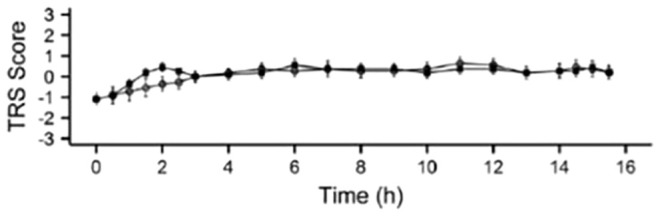
Mean (±SE) Treatment Response Scale scores (LCIG shown as filled squares; LECIG shown as open circles).^30^ Reproduced with permission from *Movement Disorders*.

To compare the pharmacokinetics of levodopa when given as LECIG or as LCIG, a randomised, open-label, 2-day, crossover trial was undertaken that measured systemic levodopa exposure over 14 h with LECIG infusion at a 20% reduced dose *versus* the patient’s usual dose of LCIG infusion.^30^ Eleven patients (aged ⩾ 30 years) with idiopathic advanced PD who had received stable LCIG treatment for at least 30 days and who had not been exposed to entacapone within 3 months of screening participated in the study. Patients were randomised to one of two treatment sequences and received either LCIG (levodopa 20 mg/ml and carbidopa monohydrate 5 mg/ml in a 100 ml cassette) followed by LECIG (levodopa 20 mg/ml, entacapone 20 mg/ml and carbidopa monohydrate 5 mg/ml in a 47 ml syringe) or LECIG followed by LCIG over two consecutive days. Both treatments were delivered *via* the same gastrojejunostomy tube *via* an infusion pump over 14 h. LECIG doses corresponding to 80% (*n* = 5) or 90% (*n* = 6) of the usual LCIG morning dose, 80% of the LCIG continuous (maintenance) dose and 80% of extra doses were administered. While the primary outcome measure was systemic exposure to levodopa, other outcomes were also evaluated, including the patient’s motor function, assessed using treatment response scale (TRS) scores;^52^ pharmacokinetics of levodopa, carbidopa, 3-O-methlydopa and entacapone; and safety by monitoring of adverse events. TRS is a 7-point scale ranging from –3 (severe parkinsonism) to 0 (‘ON’ without dyskinesia) to +3 (‘ON’ with severe dyskinesia). TRS scores from –1 to +1 are considered as functional ‘ON’ time. Despite giving a reduced levodopa dose with LECIG, systemic exposure for levodopa did not differ significantly between treatments, indicating that due to the presence of entacapone, the bioavailability of levodopa over a 14-h infusion was comparable with LECIG compared with LCIG (Figure 2).^30^ TRS scores showed no difference between treatment groups despite the lower levodopa dose administered as LECIG (Figure 3).^30^ It was concluded from this study that the increased levodopa bioavailability in the presence of entacapone meant that lower overall levodopa doses can be given with LECIG given to achieve therapeutically effective plasma concentrations. In addition, plasma 3-OMD levels increased by 22% when switching from LECIG to LCIG but decreased by 35% when switching from LCIG to LECIG. Treatment with LECIG may therefore reduce exposure to potentially harmful levodopa metabolites.

In this study, no serious or unexpected adverse events were reported and no adverse events led to discontinuation or change of therapy, although it was only undertaken over a short time period and in a small number of patients.^30^ However, the safety profile observed was in line with data from other published clinical studies of treatment with LCIG infusion that include short- and long-term multinational, randomised clinical trials and large, open-label studies with a duration of exposure of up to 4 years.^53–55^

These previous reports have shown that LCIG infusion is generally well tolerated and the safety profile consists primarily of adverse events associated with the device or surgical procedure. With long-term use, adverse events have been found to decrease over time.^47–49^ Oral entacapone has been used in combination with oral levodopa for many years in clinical practice and the safety and tolerability of this combination have been previously reported in several randomised, placebo-controlled trials with long-term, open-label extension phases.^56^ The most common dopaminergic side effects are dyskinesia and nausea, resulting from the increased bioavailability of levodopa, which can be easily managed, while nondopaminergic side effects include diarrhoea (in around 10% of patients) and harmless urine discoloration. In light of these findings, patients with known intolerance to oral entacapone may not be suitable for LECIG treatment.

Although the results of the randomised, crossover clinical trial of LECIG are positive, due to the short treatment time and the small number of patients, conclusions have to be drawn with caution.^30^ Larger comparative efficacy and safety studies are needed to confirm the results and to determine long-term outcomes and possible adverse effects. The effect of LECIG on nonmotor symptoms of PD, an important aspect of the overall PD clinical picture, has not been assessed to date.

To investigate the dose adjustment needed when switching patients from LCIG to LECIG, a population pharmacokinetic study was undertaken.^31^ A population levodopa pharmacokinetics model was developed to describe the time course of drug exposure based on plasma levodopa concentrations obtained from the 11 patients with advanced PD who had taken part in the previous randomised, crossover study of LCIG *versus* LECIG.^30^ This model was then used to simulate three alternative dose regimens for LECIG, in comparison with LCIG: (1) 100% of the LCIG morning dose and no reduction in the continuous maintenance dose, (2) a 20% lower morning dose with a 20% lower maintenance dose and (3) 100% of the morning dose with a 35% lower maintenance dose. In addition, the study evaluated the effect of common variations of the COMT and DDC genes on levodopa pharmacokinetics. Genetic variations in these genes have been suggested to affect the natural activity and/or expression of these enzymes, which in turn may affect the pharmacokinetics of levodopa. The results indicated that, on a population level, the continuous maintenance dose of LECIG should be decreased by approximately 35% to achieve similar levodopa exposure to that achieved with LCIG, while the morning dose should be maintained at 100% of the usual LCIG morning dose. Notably, this effect of entacapone on levodopa pharmacokinetics was found to occur regardless of the patient’s COMT genotype.^31^

It is likely that opicapone or tolcapone as the COMT inhibitor in the gel instead of entacapone would increase the bioavailability of levodopa even further.^50,51^ This could be an interesting approach for future drug development.

## Real-world clinical experience with LECIG infusion

LECIG infusion was approved by the Swedish Medical Products Agency in 2018 as a new DAT option and has been reimbursed in Sweden since 2019. Initial ‘real world’ clinical experience to date with LECIG infusion in Sweden has been evaluated in an observational study of 24 PD patients (12 of whom switched from LCIG infusion) undertaken at Uppsala University Hospital, Sweden. The study also described patient-reported outcomes and their perceptions of therapy.^57^ All patients with idiopathic PD treated with LECIG infusion at Uppsala University Hospital were screened for eligibility and were then followed up according to the usual clinical routine: a visit, either as outpatient or as inpatient, every 6 months, following the Swedish national guidelines for the care of PD. Patients completed questionnaires to determine their experience of any change in symptoms, the user-friendliness of the drug delivery system, patient-reported activities of daily living, patient-reported health-related quality of life and, for patients switched from LCIG to LECIG, a comparison of these two therapies. The 24 patients (11 females and 13 males) included in the analysis had a median age when starting LECIG infusion of 71.5 years, a median duration since PD diagnosis of 15.5 years and a median LECIG treatment duration of 305 days. The levodopa-equivalent dose (LED) for LECIG infusion was calculated by using the conversion factor derived by Senek *et al.*^30^ For patients switching from LCIG, at initiation, a median of 100% of the previous LCIG morning dose was used, while the continuous infusion rate with LECIG infusion was reduced to 76% of the previous LCIG dose.

Most patients reported an improvement in their ability to perform daily activities and in their self-rated quality of life with LECIG infusion treatment and a high proportion of patients (70%) who had not used any kind of levodopa infusion before (*n* = 10) perceived that their symptoms had improved. Among patients who switched from LCIG, the most common perception of the effect of LECIG infusion on PD symptoms compared with LCIG was that there was no change (45%) – this is as expected because LECIG is able to provide therapeutically effective plasma levodopa concentrations despite giving a lower overall levodopa dose. Patients also reported that they were generally happy with the user-friendliness and smaller size of the Crono^®^ LECIG pump compared with the standard LCIG pump.

LECIG infusion was approved in Germany by the Bundesinstitut für Arzneimittel und Medizinprodukte (BfArM)/Federal Institute for Drugs and Medical Devices in December 2020 and is fully reimbursed. During 2021, at the Parkinson-Klinik Ortenau in Wolfach, Germany, five patients were successfully initiated onto LECIG infusion, all of whom showed meaningful reductions in MDS-UPDRS total scores compared with baseline (prior to LECIG initiation; Table 1). The mean age of these patients when starting LECIG treatment was 71 (range = 57–82) years, the average time from initial PD diagnosis was 16 (range = 9–23) and most were assessed as being Hoehn and Yahr stage 4. Once initiated onto LECIG infusion, the morning dose ranged from 4.0 to 8.0 ml with the continuous dose ranging from 1.4 to 2.9 ml/h. All patients were connected to the pump during their waking day, usually for around 16 h, removing the pump at night. This group of patients will continue to be monitored on an ongoing basis to assess long-term efficacy and tolerability of LECIG infusion.

**Table 1.** Characteristics and LECIG dosing of patients initiated onto LECIG infusion in 2021 at the Parkinson-Klinik Ortenau, Wolfach, Germany.

Patient	Age at the start of LECIG (years)	Time since PD diagnosis (years)	Hoehn & Yahr stage	MDS-UPDRS total score		LECIG morning dose	LECIG continuous dose	LECIG bolus dose
				On hospital admission	On discharge after LECIG initiation			
**1**	66	9	4	86	61	8.0 ml	2.9 ml/h	2.0 ml
**2**	74	15	3	34	19	4.0 ml	2.4 ml/h	2.0 ml
**3**	57	13	4	96	55	6.0 ml	1.4 ml/h	1.5 ml
**4**	78	21	4	71	56	8.0 ml	1.4 ml/h	2.0 ml
**5**	81	23	4	82	55	8.0 ml	1.5 ml/h	1.0 ml

LECIG, Levodopa–entacapone–carbidopa intestinal gel; PD, Parkinson’s disease.

Collection of clinical data *via* registries provides valuable real-world data to support clinical trial evidence. Sweden has a National Parkinson’s Disease Patient Registry (PARKreg) as part of the Swedish Neuro Registry (https://www.neuroreg.se).^22^ So far, more than 100 patients on LECIG are registered in Sweden. Alongside the launch of LECIG infusion, a prospective, noninterventional, observational pan-European study has been established. The ELEGANCE study (NCT05043103) will offer participation to all patients treated with LECIG infusion as part of routine clinical practice across 16 countries. In addition to collecting data on efficacy and safety, it will also record information on patients’ quality of life and healthcare resource use.

## Practical considerations for treating patients with LECIG infusion

As with any new therapy, particularly the more complex DATs for PD, it is important that healthcare teams gain experience with the product. The aim of this section is to support this process and provide a stepwise guide for starting patients on LECIG infusion and transitioning them from other therapies.

Due to the similarities in device placement and delivery method to LCIG infusion, a multidisciplinary team (MDT) approach is important for the management of patients being treated with LECIG. Arrangements will vary between countries and centres, but the MDT will often comprise a movement disorders specialist or neurologist with expertise in movement disorders, a specialist PD nurse, a gastroenterologist or surgeon (for initial placement of the PEG-J system), and in some cases an interventional radiologist or gastroscopist (for repositioning of the tube in case of dislocation). PD nurses have a pivotal role in the MDT and are a key point of contact between the patient and the wider MDT, for monitoring them when they start therapy and on an ongoing basis, and also to educate them on the correct use of the product.

In many countries, the product manufacturer provides specialist PD nurses who can work in partnership with the healthcare team to start and monitor patients on LECIG infusion and to provide ongoing support services for patients and their families. These services may include technical helplines, product training and education resources and support materials for healthcare teams and for patients.

### Step 1: identifying advanced PD

Timely identification of patients with advanced PD who are suitable for DAT means that they can be referred promptly to movement disorders specialists and receive effective treatment. A range of guidelines and assessment tools have been developed by experienced movement disorders specialists to standardise this process and help define (1) whether the patient has advanced PD and (2) if they have, which DAT would be best suited for them.

The ‘5-2-1’ criteria were developed in 2018 by a panel of movement disorder specialists from 10 European countries to address the lack of a global consensus on the definition of advanced PD and the timing of use of DATs.^58^ ‘5-2-1’ refers to patients who receive daily oral levodopa doses ‘at least 5 times a day’, have at least 2 h of the waking day with OFF symptoms and at least 1 h of the day with troublesome dyskinesia. The consensus also provides recommendations for indicators of suitability for DATs.

NAVIGATE-PD is another tool that was developed as an educational programme to supplement existing guidelines and provide recommendations on the management of PD refractory to oral/transdermal therapies.^59^ It involved 103 experts from 13 countries and was overseen by an International Steering Committee of 13 movement disorder specialists. It compares the suitability of patients for three DATs: deep brain stimulation, subcutaneous apomorphine infusion and LCIG infusion.

MANAGE-PD (https://www.managepd.com/) is an online tool that can be used by clinicians to help identify patients inadequately controlled on oral medications.^60^

### Step 2: determining eligibility for LECIG infusion therapy

The range of DAT options should ideally be discussed with patients (and their carers) before they become candidates for these therapies. Balanced information should be given about the pros and cons of each of the different DATs along with guided discussion about which one might be best suited for them depending on their clinical picture and personal circumstances. Essentially, suitable patients for LECIG infusion are likely to be the same as those for standard LCIG infusion,^61^ except for those who have a known intolerance to entacapone. Similarly, patients who are unsuitable for LCIG treatment are likely to also be unsuitable for LECIG, including those with severe dementia or active psychosis, those who would be unable to handle the pump and PEG-J system or who do not have good carer support at home.

### Step 3: starting treatment with LECIG infusion

Ultimately, LECIG treatment involves a surgical procedure under sedation/local anaesthesia, undertaken in collaboration with the gastroenterology team, to place a permanent percutaneous endoscopic gastrostomy with jejunal tube (PEG-J). A temporary nasoduodenal/nasojejunal tube can be used initially to determine whether the patient responds favourably to this method of treatment before a permanent PEG-J system is surgically placed, but this is not undertaken at all centres. For long-term administration, LECIG infusion should be administered using the Crono^®^ LECIG pump connected to the PEG-J system that delivers the medication directly into the duodenum or upper jejunum.

LECIG infusion treatment can generally be initiated directly after an uncomplicated PEG-J placement, after consultation with the gastroenterologist. For patients transitioning from LCIG infusion to LECIG and who have an existing PEG-J system, a temporary connection adapter can be used initially to connect to the Crono^®^ LECIG pump; however, it is recommended that a permanent ENFit connector is fitted as soon as possible. A quick and simple way to transition from LCIG to LECIG infusion is to stop the LCIG pump in the evening, flush the tube as normal and then start using LECIG infusion in the morning. Alternatively, an immediate transition from LCIG to LECIG may be done anytime during the day, without flushing the tube. In such cases, LECIG may be initiated in an outpatient clinic or in a telemedicine setting.^57,62^

### Step 4: calculating the dose of LECIG infusion

A patient’s overall LED is a standard calculation obtained by adding together the LED for each antiparkinsonian drug they are taking.^63,64^ This calculation method has recently been updated to include LECIG.^65^ Calculation of the correct LECIG infusion dose is an important aspect of transitioning patients from oral medication or from LCIG infusion, but is in fact a simple two-step process, as shown below.

1. Calculate the levodopa dose required based on the patient’s current levodopa medication intake.

When transitioning to LECIG, the dose calculation should focus initially on the levodopa component of the patient’s existing medication and then adjust this to take account of the entacapone present in LECIG that allows a lower overall levodopa dose to be given. Other oral/transdermal medications that the patient is taking may then be adjusted separately as discussed in point 2 below.

As with LCIG, the total dose per day of LECIG is composed of three individually adjusted doses: the morning bolus dose, the continuous maintenance dose and extra bolus doses that may be needed throughout the day to control any recurring OFF symptoms. Suggested dose calculations when transitioning patients from oral levodopa to LECIG are shown in Table 2, and when switching patients from LCIG to LECIG are shown in Table 3. In the case of transitioning from oral levodopa, it is suggested that the initial morning dose should be decreased by 35%, and in the case of switching from LCIG it is suggested that the initial morning dose should be maintained at 100% of the previous LCIG dose. The continuous maintenance dose should be started at 65% of the previous oral levodopa or LCIG dose, based on population pharmacokinetic calculations,^31^ but 75% is a reasonable alternative from a clinical point of view, for patients who suffer from severe OFF symptoms and would prefer to start the new treatment in a dyskinetic state. The hourly dose is usually divided on 16 h of waking day, but there are individual differences in daily duration of infusion.^66^ A simpler approach to calculating the hourly maintenance dose is to determine the patient’s hourly oral levodopa dose and then divide by 20 mg/ml. For example, if a patient takes 100 mg oral levodopa (+DDCI) every 3 h, the levodopa requirement is 33.3 mg/h. Sixty-five percent of this dose, divided by 20 mg/ml equates to 1.1 ml/h of LECIG.

**Table 2.** Switching from oral levodopa^a^: an example where a patient used 100 mg of levodopa as morning dose and had a total daily dose of 1000 mg.

	Oral levodopa dose	LECIG (20 mg/ml)	
		Calculated morning dose (100% of oral dose)	Calculated continuous dose (65% of oral dose^b^)
Morning dose	100 mg	100 mg: 20 mg/ml = 5.0 ml	
Total daily dose	1000 mg		1000 mg – 100 mg = 900 mg 900 × 0.65 = 585 mg/day (29.25 ml) 29.25 ml LECIG over 16 h = 1.8 ml/h
LECIG starting dose		5.0 ml + 3.0 ml to fill the tube	1.8 ml/h

COMT, catechol-O-methyltransferase; LECIG, Levodopa–entacapone–carbidopa intestinal gel.

a These calculations assume the patient is NOT taking oral COMT inhibitors. If they are taking oral entacapone, levodopa doses do not need to be adjusted when transitioning to LECIG. Oral entacapone can be stopped once the patient commences LECIG treatment.

b The continuous maintenance dose should be based on the patient’s daily levodopa intake (excluding the morning dose) and initially reduced to 65% of the previous daily levodopa intake.

**Table 3.** Switching from standard LCIG infusion^a^: an example where a patient used 8.0 ml as morning dose and 3.0 ml/h as the continuous infusion rate.

	Previous LCIG dose	LECIG (20 mg/ml)	
		Calculated morning dose (100% of LCIG dose)^b^	Calculated continuous dose (65% of LCIG dose)
Morning dose	8.0 ml	8.0 ml	
Continuous dose	3.0 ml/h		3.0 ml × 0.65 = 1.95 ml/h
LECIG starting dose		8.0 ml (5.0 ml + 3.0 ml to fill the tube)	2.0 ml/h

COMT, catechol-O-methyltransferase; LCIG, levodopa–carbidopa intestinal gel; LECIG, Levodopa–entacapone–carbidopa intestinal gel.

a These calculations assume the patient is NOT taking oral COMT inhibitors. If they are taking oral entacapone, LCIG doses do not need to be adjusted when transitioning to LECIG. Oral entacapone can be stopped once the patient commences LECIG treatment. If they were previously taking opicapone or tolcapone, the levodopa dose should be slightly increased (see Nyholm and Jost, 2021) when switching to LECIG, while discontinuing the oral COMT inhibitor.

b The morning dose of LECIG should stay the same as that used previously for standard LCIG.

These calculations assume the patient is not taking oral COMT inhibitors. If they are taking oral entacapone, then the oral levodopa/LCIG doses do not need to be decreased when transitioning to LECIG. Once LECIG doses are calculated, they can then be used as the initial doses when commencing LECIG treatment but should be considered very much as a starting point for further adjustment and personalisation. Calculated doses may need to be titrated up or down to achieve optimum PD symptom control and each patient is likely to have slightly different dosing needs. For patients switching from LCIG, the objective should be to achieve equivalent motor symptom control with LECIG as they had previously with LCIG, but in this case using a lower levodopa dose. Morning doses and continuous doses can be fine-tuned over the course of the next few weeks according to response until optimal doses are achieved that effectively control symptoms. Extra doses do not have to be calculated. It is suggested 1.0 ml (LED 27 mg) is used initially and then individualised for the patient.

Some PD patients may require night-time LECIG infusions. Experience suggests that the night-time infusion requirement is around 50–80% of the daytime continuous infusion rate, but as for the daytime infusion rate, this may need to be adjusted to suit the needs of the individual patient. The Crono^®^ LECIG pump allows healthcare professional to programme different flow rates for daytime and night-time use, or for different flow rates during the day. The patient can then choose to manually switch between the two to three pre-programmed flow rates.

2. Adjust the dose based on clinical outcome and concomitant oral medications.

Patients at the advanced stage of PD are often taking multiple oral/transdermal PD medications, including dopamine agonists (DAs), MAO-B inhibitors and COMT inhibitors. It is important to note that when initiating patients onto any levodopa infusion therapy, the intention is not to completely replace all oral medications and give monotherapy from the outset. While this may be achievable once patients are established on treatment, it is likely that some concomitant oral medication may still be needed initially. Oral COMT inhibitors should be stopped once the patient commences LECIG treatment, as entacapone is included in the LECIG formulation. Once the patient is initiated on LECIG, gradual adjustment and down-titration of these other therapies can be commenced to find the optimum balance of efficacy and tolerability. Note that rapid or complete reduction of oral medications can be detrimental, and in the case of DAs may precipitate DA withdrawal syndrome,^67,68^ so a slow and cautious approach over a prolonged period of time is recommended to personalise and fine-tune the doses that each patient requires.

### Step 5: ongoing management of LECIG patients

Before discharge, patients also need to be able to competently carry out the daily procedures for LECIG infusion in terms of using the pump correctly, administering their morning dose, delivering their continuous dose, and know how to administer any extra doses, if needed. Certain Crono^®^ LECIG pump settings should be locked so that they can only be changed by the healthcare team to prevent accidental or unauthorised changes. These include programming of continuous infusion flow rates, setting the morning dose, and setting extra doses. It is possible to set up to three different continuous infusion flow rates, which the patient can select according to his or her condition but these need to be programmed initially by one of the healthcare team. Different flow rates during the day may be required in patients with diurnal fluctuations, which may occur in spite of the continuous infusion.^69^

As discussed previously, accumulated data for LCIG infusion show that while it is generally well tolerated, adverse events associated with the device or surgical procedure are common, particularly within the first few weeks after PEG-J placement, but generally resolve or stabilise after that time.^53–55,70^ Careful aftercare of the stoma site and PEG-J tube is therefore essential and vigilance for any adverse effects or infections is needed. Patients and their carers should be instructed about these techniques.

Other possible adverse events not related to the device or surgical procedure that healthcare teams should be aware of and monitor for are those consistent with patients receiving long-term levodopa treatment, including dyskinesias, weight loss, and the development of peripheral neuropathy.^28^ Some studies have shown that patients who developed peripheral neuropathy with LCIG infusion and who received vitamin B1 and B12 supplementation showed clinical improvement in the condition.^40^ Vitamin supplementation is therefore sometimes used in patients identified with B12 and folate deficiency prior to starting LCIG infusion, along with regular electrophysiological assessment to monitor for the development of peripheral neuropathy.^28^

## Conclusions

LECIG infusion is the newest addition to the range of DAT options and can now be considered for suitable patients alongside DBS, apomorphine infusion and LCIG infusion. Clinical study data and practical clinical experience with LECIG infusion have shown that it has similarities to LCIG infusion in terms of clinical efficacy, but the presence of entacapone in LECIG increases levodopa bioavailability, so lower overall levodopa doses can be given to achieve therapeutically effective plasma concentrations. A reduced overall levodopa dose may have the advantage of reducing exposure to potentially harmful levodopa metabolites.

Advanced PD patients who are suitable for DATs can be switched to LECIG infusion from either oral medications or other DATs using a simple, stepwise dosing calculation and titration. In the case of patients being treated with LCIG infusion who have an existing PEG-J system, this is compatible with the LECIG system, facilitating the transition. LECIG infusion also benefits from a smaller cartridge and pump system than the LCIG infusion, which patients may prefer.

Clinical experience with LECIG infusion is so far limited to 2.5 years in Sweden, where it was first launched, but real-world data will be collected and analysed on an ongoing basis in countries where LECIG infusion is introduced to monitor its long-term efficacy and safety. Future research should also specifically investigate neuropathy development and health-related quality of life associated with the treatment. Furthermore, the cost-effectiveness of LECIG in different patient populations should be evaluated to further understand the value of this relatively new therapy.
